# Evaluation of CAR-T Cells’ Cytotoxicity against Modified Solid Tumor Cell Lines

**DOI:** 10.3390/biomedicines11020626

**Published:** 2023-02-19

**Authors:** Aigul Kh. Valiullina, Ekaterina A. Zmievskaya, Irina A. Ganeeva, Margarita N. Zhuravleva, Ekaterina E. Garanina, Albert A. Rizvanov, Alexey V. Petukhov, Emil R. Bulatov

**Affiliations:** 1 Institute of Fundamental Medicine and Biology, Kazan Federal University, 420008 Kazan, Russia; 2 Institute of Hematology, Almazov National Medical Research Center, 197341 Saint Petersburg, Russia; 3 Shemyakin-Ovchinnikov Institute of Bioorganic Chemistry, Russian Academy of Sciences, 117997 Moscow, Russia

**Keywords:** CAR-T, cytotoxicity, solid tumor, tumor model, cytokine, chemokine

## Abstract

In recent years, adoptive cell therapy has gained a new perspective of application due to the development of technologies and the successful clinical use of CAR-T cells for the treatment of patients with malignant B-cell neoplasms. However, the efficacy of CAR-T therapy against solid tumor remains a major scientific and clinical challenge. In this work, we evaluated the cytotoxicity of 2nd generation CAR-T cells against modified solid tumors cell lines—lung adenocarcinoma cell line H522, prostate carcinoma PC-3M, breast carcinoma MDA-MB-231, and epidermoid carcinoma A431 cell lines transduced with lentiviruses encoding red fluorescent protein Katushka2S and the CD19 antigen. A correlation was demonstrated between an increase in the secretion of proinflammatory cytokines and a decrease in the confluence of tumor cells’ monolayer. The proposed approach can potentially be applied to preliminarily assess CAR-T cell efficacy for the treatment of solid tumors and estimate the risks of developing cytokine release syndrome.

## 1. Introduction

Adoptive cell therapy (ACT) is a cornerstone of contemporary immunotherapy in oncology. One of the most important branches of ACT, chimeric antigen receptor (CAR-T) therapy, has demonstrated remarkable efficacy against hematological cancers [1]. There are six CAR-T drugs now approved by the FDA: Kymriah, Yescarta, Tecartus, Breyanzi, Abecma, and Carvykti. Despite the fact that numerous clinical trials of ACT against solid tumors are currently underway, there are still significant hurdles in the treatment of solid tumors (NCT03198052, NCT04652219, NCT04348643, etc.). Sadly, the results of completed trials indicate a short duration of therapeutic effect, if any [2].

Such results have several objective causes. First, there is a difficulty with antigen heterogeneity and a finite number of targetable antigens. Chemokine dysregulation, aberrant vascularization, and thick tumor stroma are additional factors that obstruct T cell migration into the tumor site. Moreover, even when T cells reach their destination, they encounter unfavorable conditions, such as a lack of oxygen and nutrition, a low pH, and an immunosuppressive tumor microenvironment, making T cell survival extremely improbable [3].

In addition to serious life-threatening adverse effects, the administration of CAR-T therapy against solid tumors is hindered by cytokine release syndrome (CRS), which presents an avalanche-like production of proinflammatory cytokines by hyperactive immune cells. The symptoms of CRS include fever, hypotension, shock, and organ toxicity [4]. The in vitro prediction of probable CRS development during CAR-T therapy remains an unresolved issue.

Nevertheless, these challenges are not intractable, and a large number of researchers are attempting to strengthen the CAR-T cell approach against solid tumors [3,5,6]. For that, the design of suitable and precise preclinical testing of CAR-T efficacy and safety using in vitro models is essential. In addition, the involvement of each pathway in tumor resistance for various forms of cancer must be defined in order to maximize efficiency.

The current study examines the efficacy of CAR-T cells against modified CD19+ solid tumor cell models. Four types of tumor cell cultures were used: lung adenocarcinoma (H522), prostate carcinoma (PC-3M), epidermoid carcinoma (A431), and triple-negative breast carcinoma (MDA-MB-231). The study was carried out with a monolayer and more complex three-dimensional models generated using a 3D bioprinter. The use of different types of common solid tumor cells to assess the effectiveness of CAR-T cells is explained by a need to explore the potentially different antitumor responses towards them. In addition, we evaluated the relevance of tumor cell line characteristics to developing resistance towards CAR-T cell treatment.

## 2. Materials and Methods

### 2.1. Obtaining CAR-T Cells

To obtain lentiviral vector particles, the construct encoding CAR receptor was transfected into HEK293T cells, together with the packaging plasmid PsPax2 (Addgene plasmid #12260) and the envelope plasmid pMD2.G (Addgene plasmid #12259) using the transfecting agent PEI MAX (Polysciences, Warrington, PA, USA). After incubation for 12 h, the medium was replaced with DMEM containing glutamine (Thermofisher, Waltham, MA, USA) with the addition of 5% fetal bovine serum (FBS) (Hyclone, Logan, UT, USA), 5 mmol sodium butyrate, and incubated further for another 28 h. The supernatant containing viral particles was collected and filtered using a sterile membrane filter with a pore diameter of 0.45 μm (Millipore, Burlington, MA, USA). Then, concentration was carried out with a centrifugal module Amicon Ultra-15, 100 kDa (Millipore, Burlington, MA, USA) at 3000 × *g* for 30 min. Subsequently, mononuclear cells of peripheral blood were isolated by centrifugation using Ficoll gradient (PanEco, Moscow, Russia), according to approval protocol N27 (28.12.2020) of the Local Ethics Committee of Kazan Federal University. The cells were cultured at 1 × 10^6^ cells/mL in RPMI-1640 medium (PanEco, Russia) containing L-glutamine (PanEco, Russia), penicillin/streptomycin (PanEco, Russia), and 10% fetal bovine serum (FBS) (Hyclone, USA).

Dynabeads Human T-activator CD3/CD28 (Invitrogen, Carlsbad, CA, USA) immunomagnetic particles were used for the selection and activation of T-lymphocytes at a ratio of 2 particles per cell. A subpopulation of T-lymphocytes was cultured in RPMI-1640 medium (PanEco, Russia) supplemented with 10% FBS (Hyclone, USA), 100 U/mL penicillin, 100 μg/mL streptomycin, and 300 U/mL recombinant human interleukin 2 (IL-2) (Biotech, Moscow, Russia). Forty-eight hours after activation, the T-lymphocytes were subjected to viral transduction under conditions of multiple infection using 50 μg/mL protamine sulfate (Sigma-Aldrich, Louis, MO, USA). After transduction, T cells continued to be incubated in RPMI-1640 medium (PanEco, Russia) for another 72 h. Further, the transduction efficiency of T-lymphocytes by lentivirus was assessed by flow cytometry [7].

### 2.2. Obtaining a Solid Tumor Cell Line Expressing the Red Fluorescent Protein Katushka2S and CD19 Antigen

#### 2.2.1. Obtaining Lentiviral Particles

HEK293T cells were simultaneously transfected with a plasmid carrying pKatushka2S transgene (FP762b, Evrogen, Moscow, Russia), packaging plasmid PsPax2 (Addgene plasmid #12260), and envelope plasmid pCMV-VSV-G (Addgene plasmid #8454). Lentiviral particles were collected from the supernatant of transfected cells and concentrated using Optima L-90K ultracentrifuge using Ultra-Clear Tubes 1 × 31/2 in. (25 × 89 mm) and a SW28 rotor (Beckman Coulter Inc., Brea, CA, USA). Concentrated viral particles were transferred to tubes and frozen at −80 °C.

#### 2.2.2. Cell Transduction

Lung adenocarcinoma H522 (ATCC# CRL-5810), prostate carcinoma PC-3M (ATCC# CRL-1435), epidermoid carcinoma A431 (ATCC# CRL-1555), and triple-negative breast carcinoma MDA-MB-231 (ATCC# HTB-26) cells were seeded in a 12-well plate at 6 × 10^4^ cells per well. The cells were cultured in a CO_2_ (ESCO, Horsham, PA, USA) incubator for 16–20 h, after which 150 μL of concentrated lentivirus was added to 350 μL of cell culture medium. After 16 h of incubation, the medium was changed for fresh growth medium. The transduction efficiency was assessed after 48 h using a CytoFLEX S flow cytometer (Beckman Coulter Inc., Brea, CA, USA) to confirm generation of H522(Kat+), PC-3M(Kat+), A431(Kat+), and MDA-MB-231(Kat+) cell lines. After that, some of the modified cells were transduced with lentivirus encoding the CD19 antigen. The transduction efficiency was assessed after 48 h using a CytoFLEX S flow cytometer (Beckman Coulter Inc., USA) to confirm generation of H522(Kat+CD19+), PC-3M(Kat+CD19+), A431(Kat+CD19+), and MDA-MB-231(Kat+CD19+) cell lines. In addition, H522(CD19+), PC-3M(CD19+), A431(CD19+), and MDA-MD-231(CD19+) lines were obtained by lentiviral transduction for further use in real-time biosensor cell analysis with xCELLigence system.

### 2.3. Evaluation of CAR-T Cells’ Cytotoxicity against Generated CD19+ Tumor Cells Using xCELLigence Biosensor Analyzer

Analysis of the proliferative activity of H522, PC-3M, A431, and MDA-MB-231, as well as their CD19+ modifications in real-time mode, was carried out using xCELLigence biosensor cell analyzer (ACEA Biosciences, San Diego, CA, USA). To monitor proliferation, tumor cells were seeded on E-plate 16 (ACEA Biosciences, USA) at 5 × 10^3^ cells per well in RPMI-1640 culture medium and incubated for 24 h. After that, CAR-T or T (control) cells were added to the appropriate wells, the incubation was continued until complete elimination of H22(CD19+), PC-3M(CD19+), A431(CD19+), and MDA-MB-231(CD19+) cell lines. The measurements of the cell index were conducted automatically every 15 min.

The cell index is a parameter that depends on the change in electrical resistance of the electrode at the well bottom and is directly proportional to the number of cells attached to the plate at a given time. Thus, by recording the readings of the device every 15 min, curves were obtained that reflect the rate of cell proliferation in real-time [8].

### 2.4. Evaluation of CAR-T Cells Efficacy against H522 and PC-3M Tumor Cells

To assess the ability to destroy H522(Kat+), PC-3M(Kat+), A431(Kat+), MDA-MB-231(Kat+), H522(Kat+CD19+), PC-3M(Kat+CD19+), A431(Kat+CD19+), and MDA-MB-231(Kat+CD19+) tumor cells, CAR-T cells were seeded on a culture plate at 5 × 10^4^ cells per well. After 12 h, CAR-T cells at 1 × 10^5^ cells per well were added and incubation continued. Then, the cell cultures were dynamically observed using a confocal scanning microscope LSM 700 (Carl Zeiss, Oberkochen, Germany). At least 6 representative micrographs of the studied cell cultures were recorded on days 2, 5, and 7 of cultivation. Micrographs were processed using the ImageJ software—the percentage of the area occupied by PC-3M(Kat+), H522(Kat+), A431(Kat+), MDA-MB-231(Kat+), PC-3M(Kat+CD19+), H522(Kat+CD19+), A431((Kat+CD19+), and MDA-MB-231(Kat+CD19+) cells was calculated. To determine the statistical significance of the differences in the monolayer densities on various days of cultivation, the Kruskal–Wallis test and the SPSS v17.0 software were used.

### 2.5. Evaluation of the Effectiveness of CAR-T Cells against 3D Tumor Cell Cultures

To assess the ability of CAR-T cells to penetrate deep into three-dimensional tumor-like structures, a three-dimensional in vitro tumor model was generated in the form of a cylinder, 5 mm wide and 3 mm high. The model was designed using Blender software, converted to gcode format using slic3r, and adapted for printing in a 96-well plate format using Sublime Text 3 software. A three-dimensional in vitro tumor model was bioprinted with Inkredible bioprinter (CELLINK, Göteborg, Sweden) using bioink CELLINK A-RGD (CELLINK, Sweden) according to manufacturer’s protocol. H522(Kat+), H522 (Kat+CD19+), PC-3M(Kat+), PC-3M(Kat+CD19+), A431(Kat+), A431(Kat+CD19+), MDA-MB-231(Kat+), and MDA-MB-231(Kat+CD19+) cells were used at 1 × 10^7^ cells per ml to prepare the bioink composition.

After 24 h of cultivation, CAR-T cells were added to the culture medium at 1 × 10^6^ cells per well. Every day, 50% of the culture medium was replaced with fresh one. The collected medium was subsequently used for multiplex analysis of cytokines and chemokines.

To assess the nature of the interaction between CAR-T cells and tumor-like structures, daily dynamic observation was performed using confocal scanning microscope LSM 700 (Carl Zeiss, Oberkochen, Germany) in 3D mode. The penetration depth of CAR-T cells was assessed by analysis of fluorescence micrographs and cross-sections of 3D cultures of tumor cells stained with hematoxylin and eosin using the Las Ez 4.0 program. One-way analysis of variance was used to determine the statistical significance of differences in penetration depths of CAR-T cells into 3D tumor cell structures (within one cultivation period).

### 2.6. Multiplex Analysis of Supernatants for Cytokines

All supernatants after co-incubation of cells were collected for subsequent multiplex analysis for cytokines using the Bio-Plex Pro Human Cytokine Panel kit, 17-plex assay (Bio-Rad, Hercules, CA, USA) on a Bio-Plex 200 (Bio-Rad, USA) according to manufacturer’s protocol.

## 3. Results

### 3.1. Preparation and Phenotyping of CAR-T Cells

**Figure 1:**
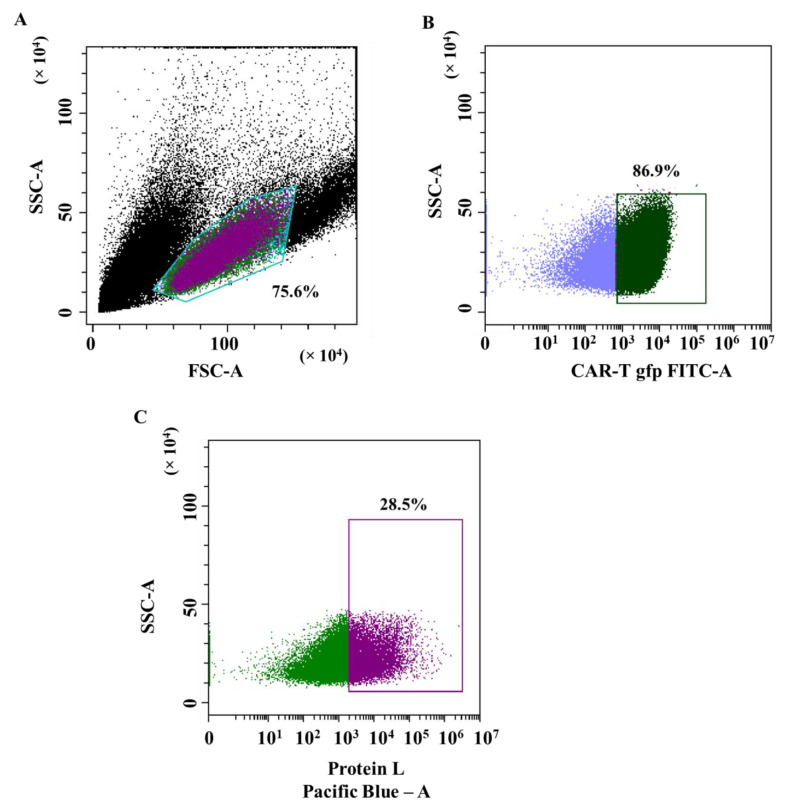
Evaluation of T-lymphocytes transduction efficiency by lentiviral particles. (**A**)—dot plot: T cell population on the forward and side scatter plot; (**B**)—dot plot: population of GFP+ transduced T-lymphocytes (CAR-T cells); and (**C**)—dot plot: population of CAR+ T cells (CAR-T cells) in GFP+ population assessed via biotinylated protein L bound to streptavidin-Pacific Blue conjugate.

The lentiviral transduction efficiency of T-lymphocytes was assessed by the signal of the GFP reporter protein using flow cytometry. Figure 1A shows a graph of forward and side scatter; a population of T cells is equal to 75.6%. The lentiviral transduction efficiency was equal to 86.98% (the proportion of cells with GFP fluorescence) (Figure 1B). The efficiency of T-lymphocyte transduction, estimated by protein L, was equal to 28.5% (Figure 1C).

### 3.2. Generating Solid Tumor Cell Lines Expressing Red Fluorescent Protein Katushka2S and CD19 Antigen

**Figure 2:**
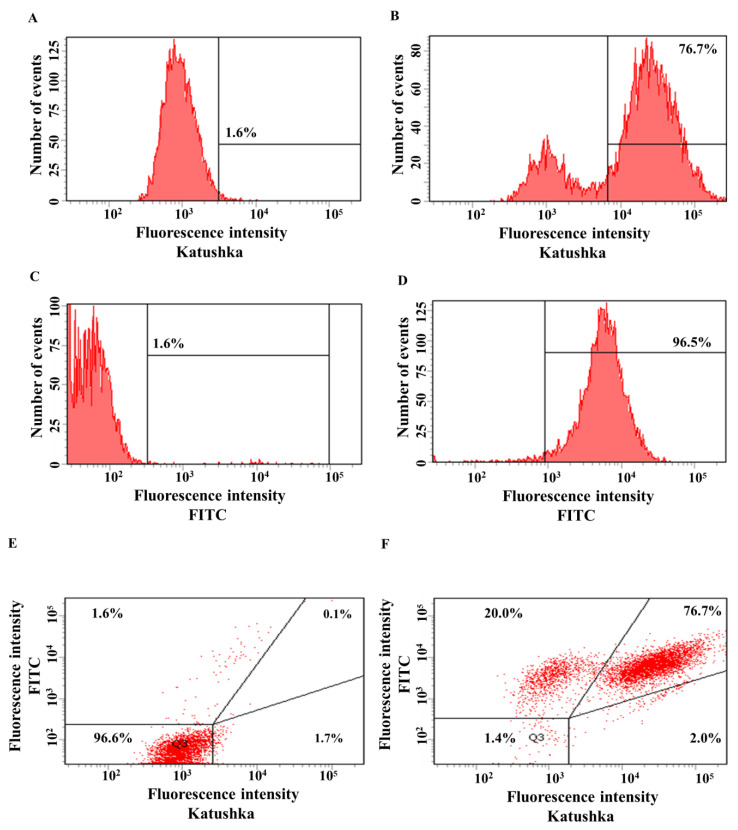
Evaluation of the transduction efficiency of H522 cells with lentiviruses encoding Katushka2S and/or CD19. Flow cytometry, representative histograms (**A**–**D**), and dot plots (**E**,**F**) are presented. (**A**)—negative control, non-transduced cells; (**B**)—H522(Kat+) cells expressing red fluorescent protein Katushka2S; (**C**)—negative control, non-transduced cells; (**D**)—H522(CD19+) cells expressing the CD19 antigen; (**E**)—negative control, non-transduced cells are shown in the upper right corner; and (**F**)—H522(Kat+CD19+) cells simultaneously expressing both red fluorescent protein Katushka2S and the CD19 antigen are shown in the upper right corner.

The transduction efficiency of the human lung adenocarcinoma cell line H522 with lentivirus encoding red fluorescent protein Katushka was equal to 76.7%, while the control sample (non-transduced cells) showed 1.6% of background fluorescence (Figure 2A,B). The transduction efficiency of H522 cells with lentivirus encoding the CD19 antigen was equal to 96.5%, while the control sample (non-transduced cells)—1.6% of background fluorescence (Figure 2C,D). H522(Kat+) cells were also transduced with lentivirus encoding CD19 to obtain H522(Kat+CD19+) cells with 76.7% purity, the control sample (non-transduced cells)—0.1% (Figure 2E,F).

**Figure 3:**
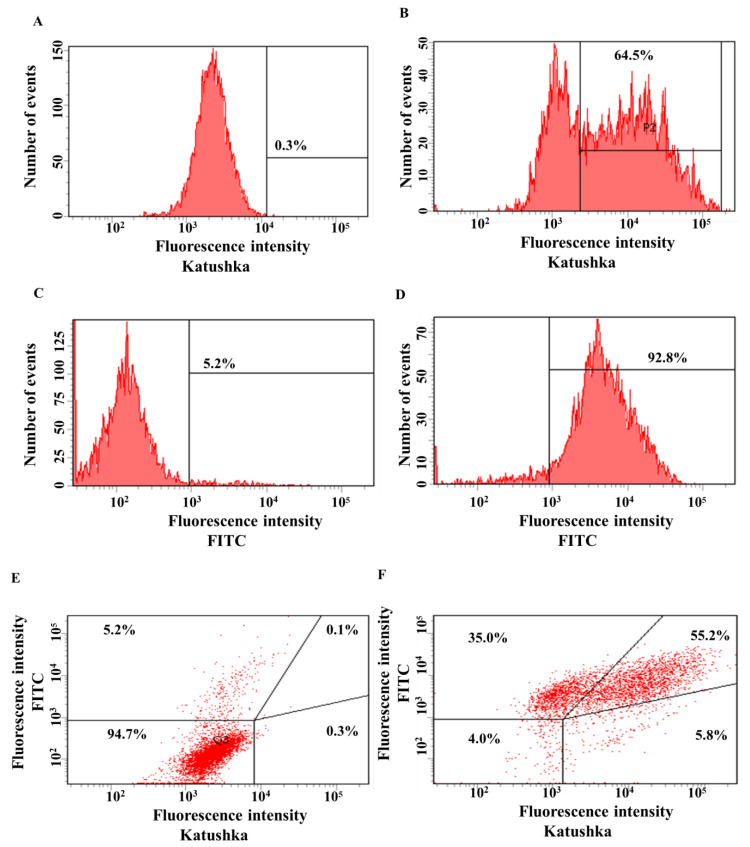
Evaluation of the transduction efficiency of PC-3M cells by lentiviruses encoding Katushka2S and/or CD19. Flow cytometry, representative histograms (**A**–**D**), and scatter plots (**E**,**F**) are presented. (**A**)—negative control, non-transduced cells; (**B**)—PC-3M(Kat+) cells expressing red fluorescent protein Katushka2S; (**C**)—negative control, non-transduced cells; (**D**)—PC-3M(CD19+) cells expressing CD19 antigen; (**E**)—negative control, non-transduced cells are shown in the upper right corner; and (**F**)—PC-3M(Kat+CD19+) cells expressing both red fluorescent protein Katushka2S and CD19 antigen are shown in the upper right corner.

The transduction efficiency of the PC-3M cell line with lentivirus encoding red fluorescent protein Katushka2S was equal to 64.5%, while the control sample (non-transduced cells) showed 0.3% of background fluorescence (Figure 3A,B). The transduction efficiency of the PC-3M cell line with the lentivirus encoding the CD19 antigen was 92.8%, and the control sample (non-transduced cells)—5.2% (Figure 3C,D). PC-3M(Kat+) cells were also transduced with lentivirus encoding CD19 to obtain PC-3M(Kat+CD19+) cells with 55.2% purity, the control sample (non-transduced cells)—0.1% (Figure 3E,F).

**Figure 4:**
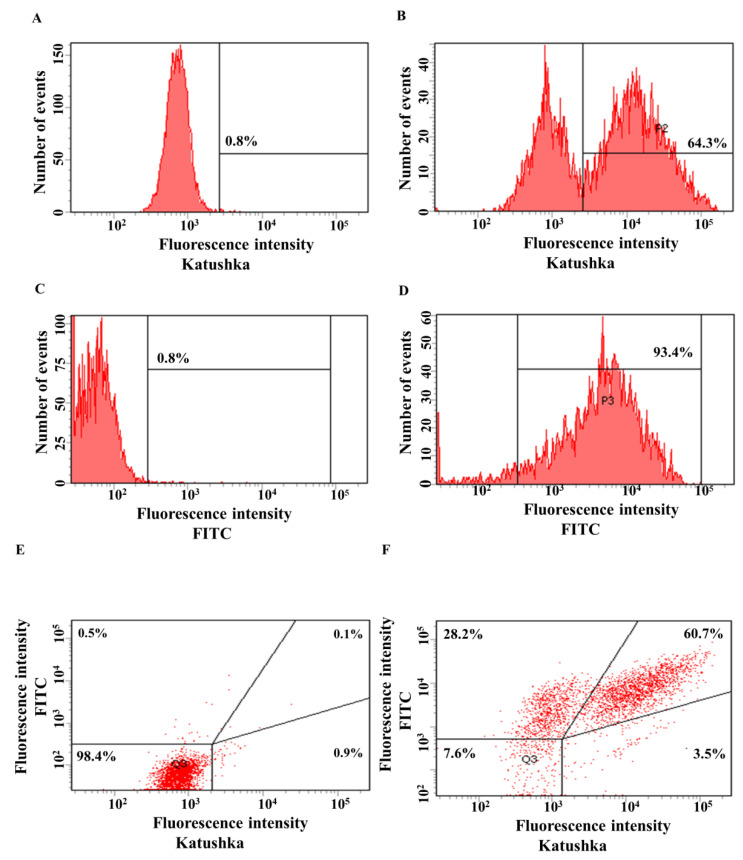
Evaluation of the transduction efficiency of A431 cells by lentiviruses encoding Katushka2S and/or CD19. Flow cytometry, representative histograms (**A**–**D**), and scatter plots (**E**,**F**) are presented. (**A**)—negative control, non-transduced cells; (**B**)—A431(Kat+) cells expressing red fluorescent protein Katushka2S; (**C**)—negative control, non-transduced cells; (**D**)—A431(CD19+) cells expressing CD19 antigen; (**E**)—negative control, non-transduced cells are shown in the upper right corner; and (**F**)—A431(Kat+CD19+) cells simultaneously expressing Katushka2S and CD19 are shown in the upper right corner.

The efficiency of A431 transduction by lentivirus encoding red fluorescent protein Katushka2S was 64.3%, in the control sample (non-transduced cells)—0.8% (Figure 4A,B). The efficiency of transduction of A431 cells with lentivirus encoding the CD19 antigen was 93.4%, in the control sample (non-transduced cells)—0.8% (Figure 4C,D). A431(Kat+) cells were also transduced with lentivirus encoding CD19 to obtain A431(Kat+CD19+) cells with 60.7% purity, the control sample (non-transduced cells)—0.1% (Figure 4E,F).

**Figure 5:**
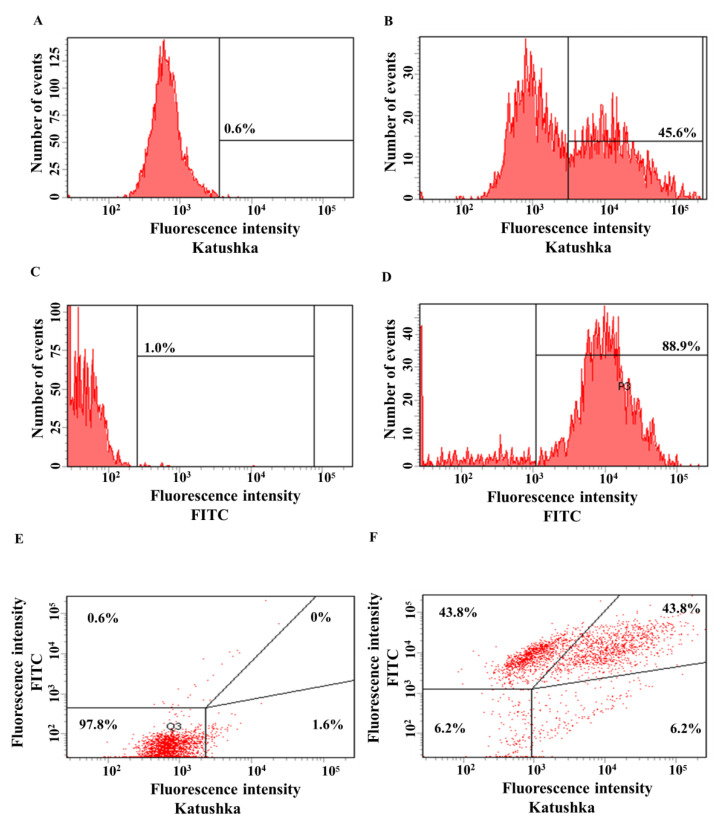
Evaluation of the transduction efficiency of MDA-MB-231 cells by lentiviruses encoding red fluorescent protein Katushka2S and/or CD19 antigen. Flow cytometry, representative histograms (**A**–**D**), and scatter plots (**E**,**F**) are presented. (**A**)—negative control, non-transduced cells; (**B**)—MDA-MB-231(Kat+) cells expressing Katushka2S; (**C**)—negative control, non-transduced cells; (**D**)—MDA-MB-231(CD19+) cells expressing CD19; (**E**)—negative control, non-transduced cells are shown in the upper right corner; and (**F**)—MDA-MB-231(Kat+CD19+) cells expressing both Katushka2S and CD19 are shown in the upper right corner.

The transduction efficiency of MDA-MB-231 cells with the lentivirus encoding Katushka2S was 45.6%, in the control sample (non-transduced cells)—0.6% (Figure 5A,B). The transduction efficiency of MDA-MB-231 cells by lentivirus encoding the CD19 antigen was 88.9%, in the control sample (non-transduced cells)—1.0% (Figure 5C,D). MDA-MB-231(Kat+) cells were also transduced with lentivirus encoding CD19 to obtain MDA-MB-231(Kat+CD19+) cells with 43.8% purity, the control sample (non-transduced cells)—0% (Figure 5E,F).

### 3.3. Evaluation of the Cytotoxicity of CAR-T Cells against Modified CD19+ Solid Tumor Cells Using xCELLigence Real-Time Cell Analyzer

**Figure 6:**
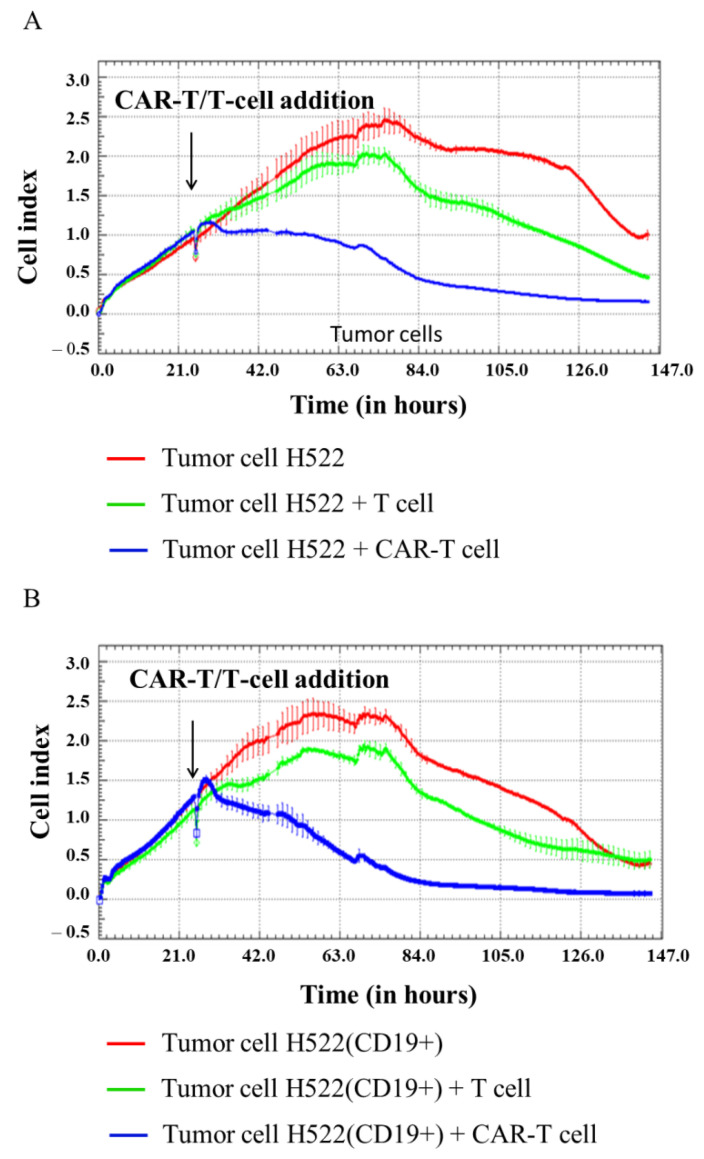
Evaluation of the cytotoxicity of CAR-T cells against H522 and H522(CD19+) tumor cell lines using xCELLigence biosensor cell analyzer. Dynamic monitoring of proliferation of H522 and modified H522(CD19+) cells with and without the addition of T/CAR-T cells. Graphs of cell index versus time are presented as mean, the error bars indicate standard deviation (*n* = 3). (**A**)—H522 cells; (**B**)—H522(CD19+) cells.

**Figure 7:**
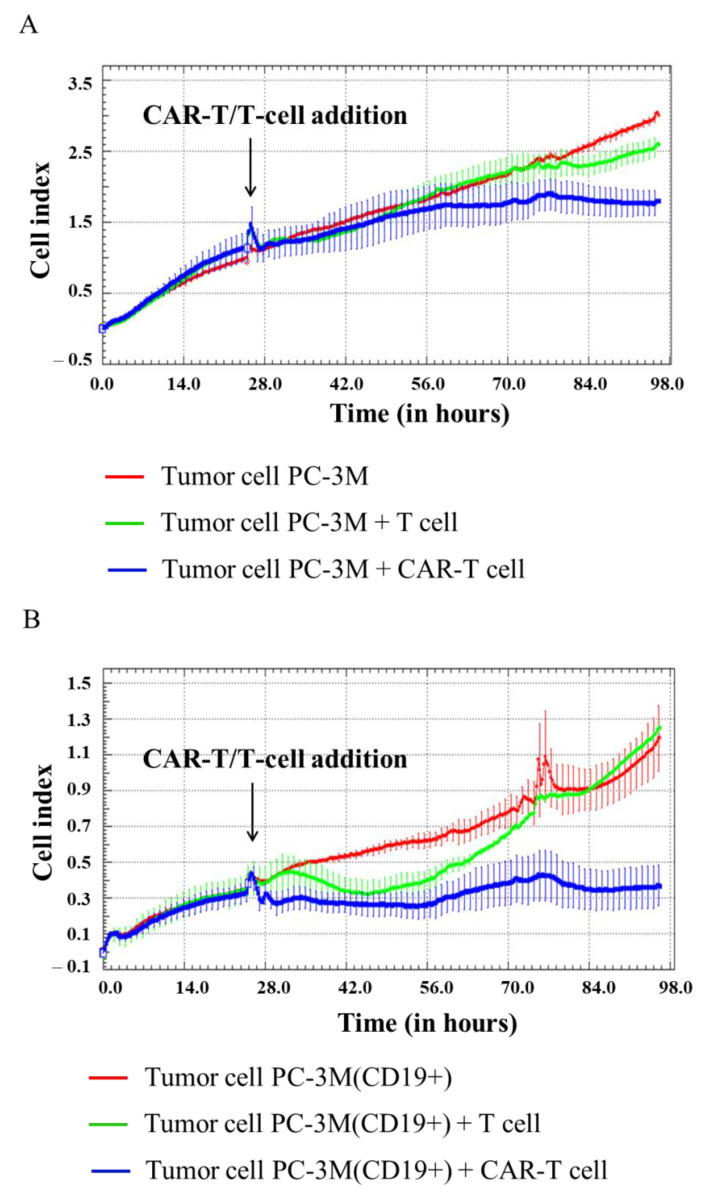
Evaluation of the cytotoxicity of CAR-T cells against PC-3M and PC-3M(CD19+) tumor cell lines using xCELLigence biosensor cell analyzer. Dynamic monitoring of proliferation of PC-3M and modified PC-3M(CD19+) cells with and without the addition of T/CAR-T cells. Graphs of cell index versus time are presented as mean, the error bars indicate standard deviation (*n* = 3). (**A**)—PC-3M cells; (**B**)—PC-3M(CD19+) cells.

**Figure 8:**
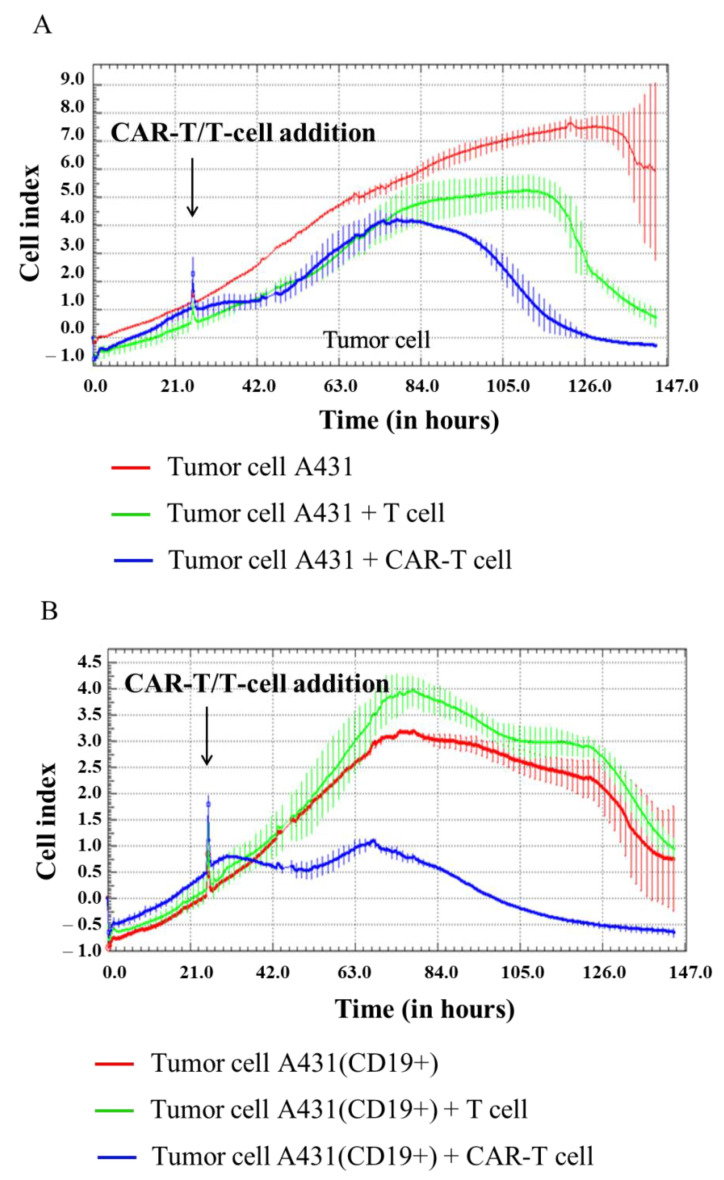
Evaluation of the cytotoxicity of CAR-T cells against A431 and A431(CD19+) tumor cell lines using xCELLigence biosensor cell analyzer. Dynamic monitoring of proliferation of A431 and modified A431(CD19+) cells with and without the addition of T/CAR-T cells. Graphs of cell index versus time are presented as mean, the error bars indicate standard deviation (*n* = 3). (**A**)—A431 cells; (**B**)—A431(CD19+) cells.

**Figure 9:**
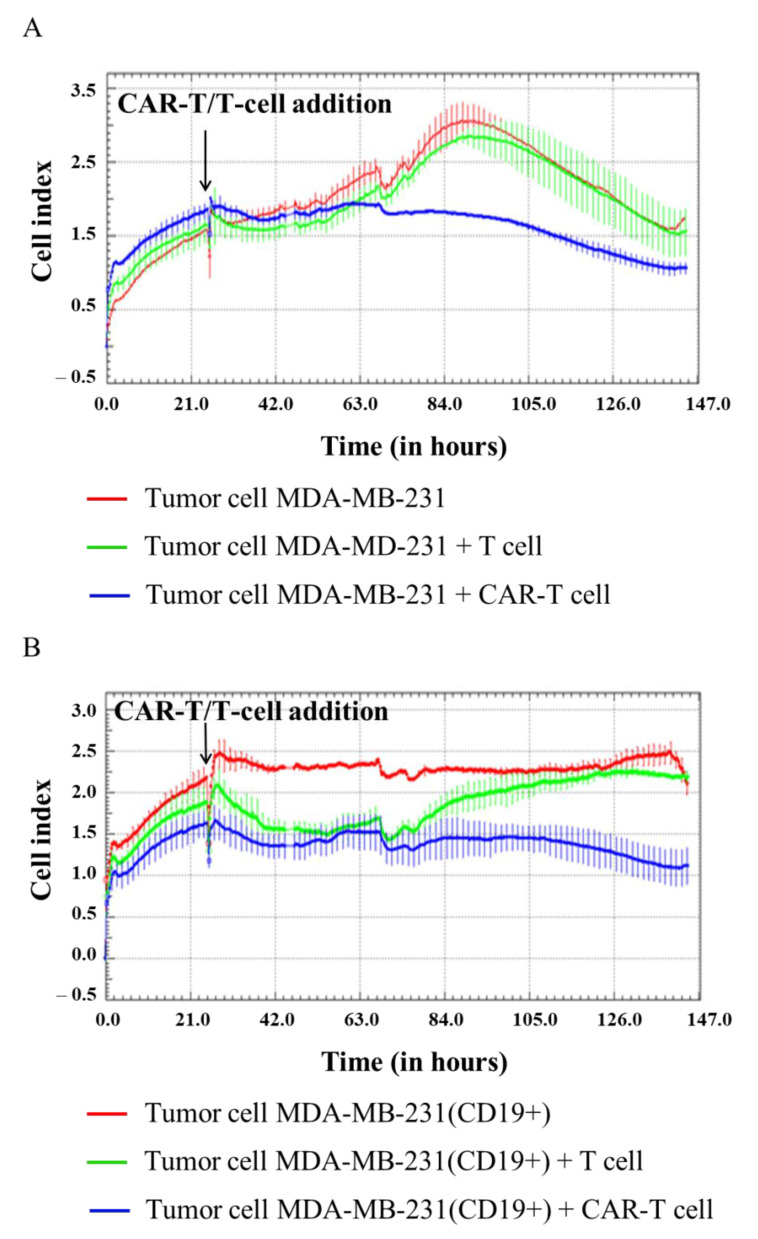
Evaluation of the cytotoxicity of CAR-T cells against MDA-MB-231 and MDA-MB-231(CD19+) tumor cell lines using xCELLigence biosensor cell analyzer. Dynamic monitoring of proliferation of MDA-MB-231 and modified MDA-MB-231(CD19+) cells with and without the addition of T/CAR-T cells. Graphs of cell index versus time are presented as mean, the error bars indicate standard deviation (*n* = 3). (**A**)—MDA-MB-231 cells; (**B**)—MDA-MB-231(CD19+) cells.

In our experiment, H522, H522(CD19+), PC-3M, PC-3M(CD19+), A431, A431(CD19+), MDA-MB-231, and MDA-MB-231(CD19+) tumor cell lines were seeded at 5 × 10^4^ cells per well in an E-plate, and after 24 h 1 × 10^5^ CAR-T cells were added per well. Then, cell proliferation was monitored using the xCELLigence biosensor system. The analysis revealed a significant decrease in proliferative activity of H522(CD19+) PC-3M(CD19+), A431(CD19+), and MDA-MB-231(CD19+) tumor cell lines after adding CAR-T cells compared to control non-modified H522 and PC-3M cells. Interestingly, a diminished proliferation of H522(CD19+), PC-3M(CD19+), A431(CD19+), and MDA-MB-231(CD19+) cells was also observed after the addition of T cells (Figure 6, Figure 7, Figure 8 and Figure 9). This might indicate non-specific partial cytolysis of CD19+ tumor cells.

### 3.4. Evaluation of the Effectiveness of CAR-T Cells against Tumor Cell Lines

**Figure 10:**
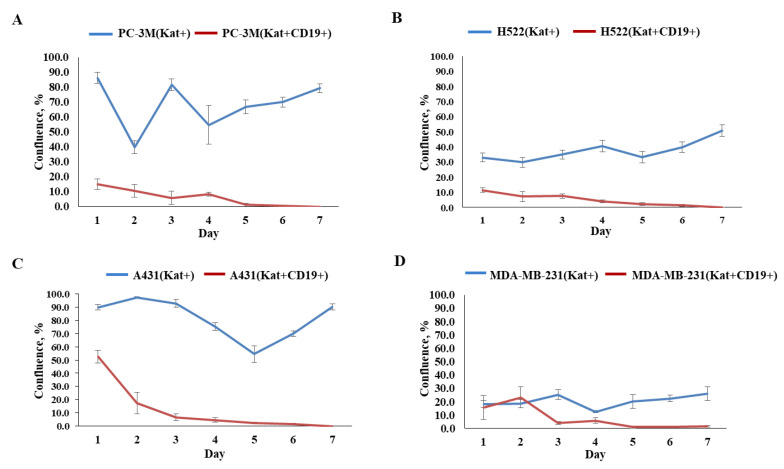
Graphs representing comparative evaluation of CAR-T cells’ efficacy against modified tumor cells over 7-day period. (**A**)—comparative confluence of PC-3M(Kat+) and PC-3M(Kat+CD19+) cells after addition of CAR-T cells; (**B**)—comparative confluence of H522(Kat+) and H522(Kat+CD19+) cells after addition of CAR-T cells; (**C**)—comparative confluence of A431(Kat+) and A431(Kat+CD19+) cells after addition of CAR-T cells; (**D**)—comparative confluence of MDA-MB-231(Kat+) and MDA-MB-231(Kat+CD19+) cells after addition of CAR-T cells. The results are presented as mean, the error bars indicate standard deviation (*n* = 3, *p* < 0.05). Control tumor cells are shown in blue, modified CD19+ tumor cell lines in red.

Graphical analysis of the fluorescent micrographs by dynamic observation of H522(Kat+CD19+), PC-3M(Kat+CD19+), A431(Kat+CD19+), and MDA-MB-231(Kat+CD19+) cells treated with CAR-T cells revealed a significant progressive decrease in the confluence of the tumor cell monolayers (Figure 10). At the same time, in the control H522(Kat+), PC-3M(Kat+), A431(Kat+), and MDA-MB-231(Kat+CD19+) cells the addition of CAR-T cells did not result in a consistent reduction in the tumor cells’ confluence throughout the entire period of cultivation (7 days). The graphical analysis of micrographs was carried out using ImageJ software.

**Figure 11:**
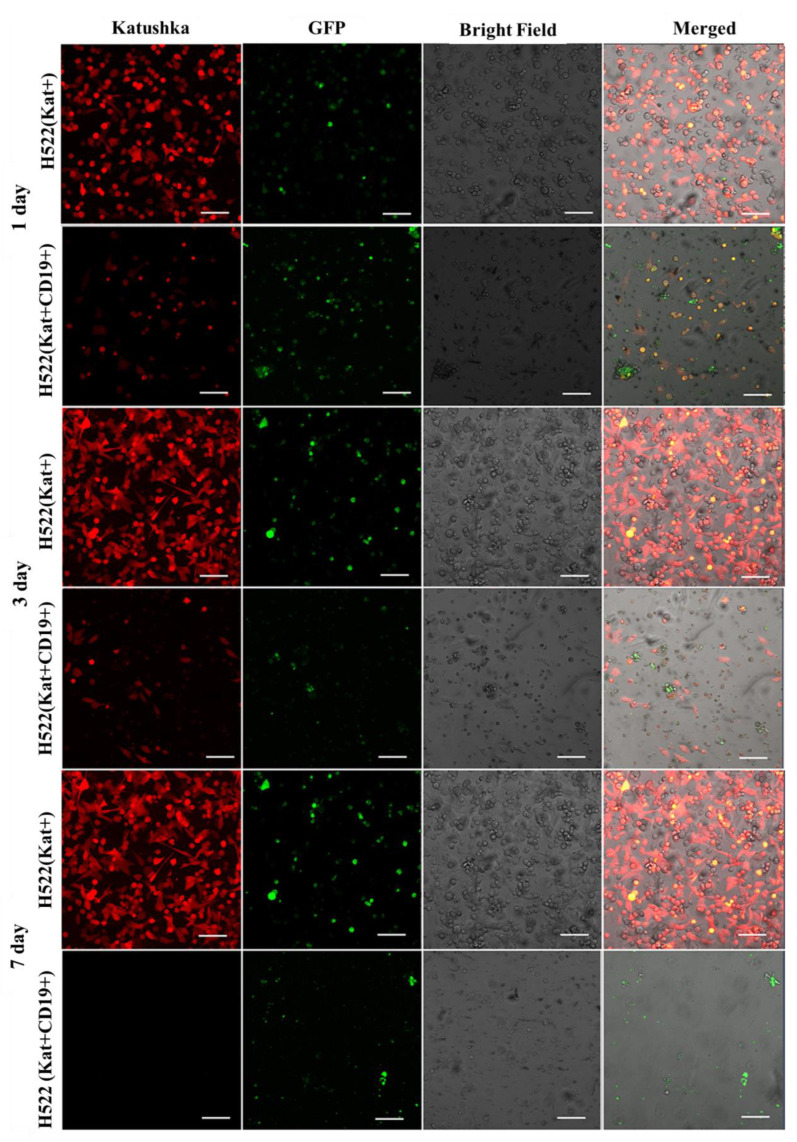
Micrographs of H522(Kat+) and H522(Kat+CD19+) tumor cell monolayers treated with CAR-T cells for a period of 7 days. Light microscopy, fluorescence microscopy, and representative micrographs (*n* = 6) are presented, scale bar is equal to 100 µm. CAR-T cells—green fluorescence; H522(Kat+) and H522(Kat+CD19+) cells—red fluorescence.

**Figure 12:**
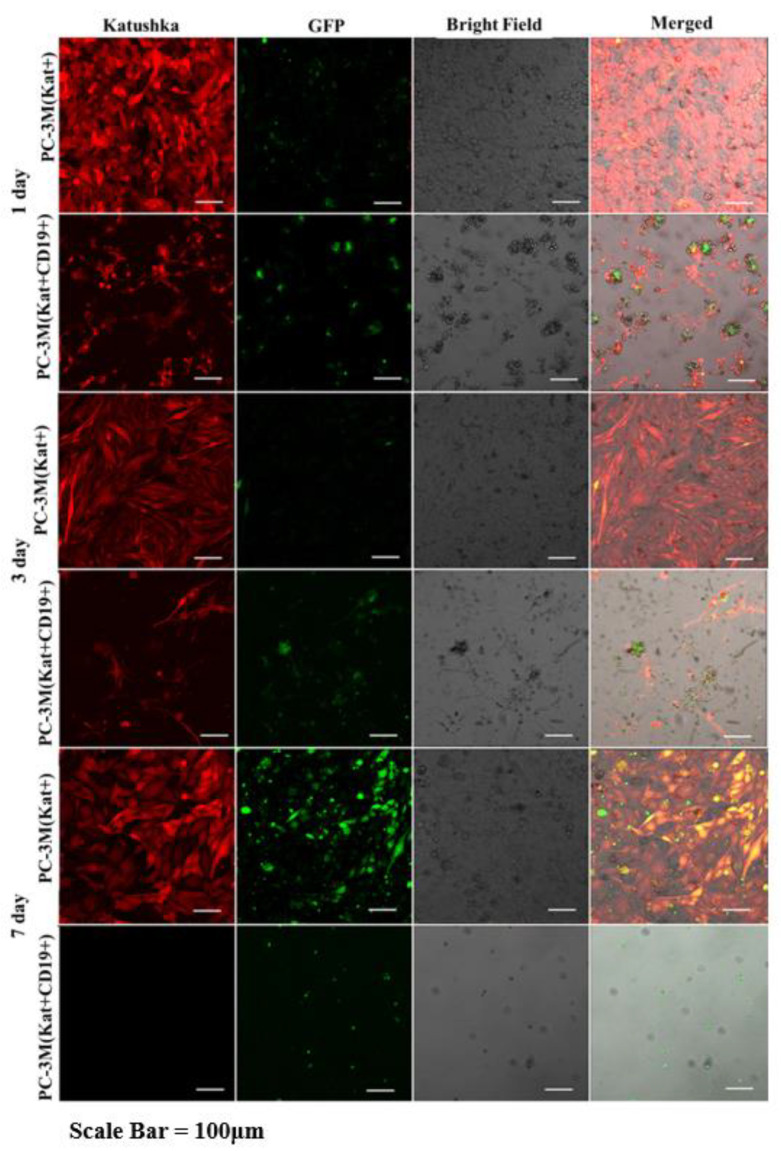
Micrographs of PC-3M(Kat+) and PC-3M(Kat+CD19+) tumor cell monolayers treated with CAR-T cells for a period of 7 days. Light microscopy, fluorescence microscopy, and representative micrographs (*n* = 6) are presented, scale bar is equal to 100 µm. CAR-T cells—green fluorescence; PC-3M(Kat+) and PC-3M(Kat+CD19+) cells—red fluorescence.

**Figure 13:**
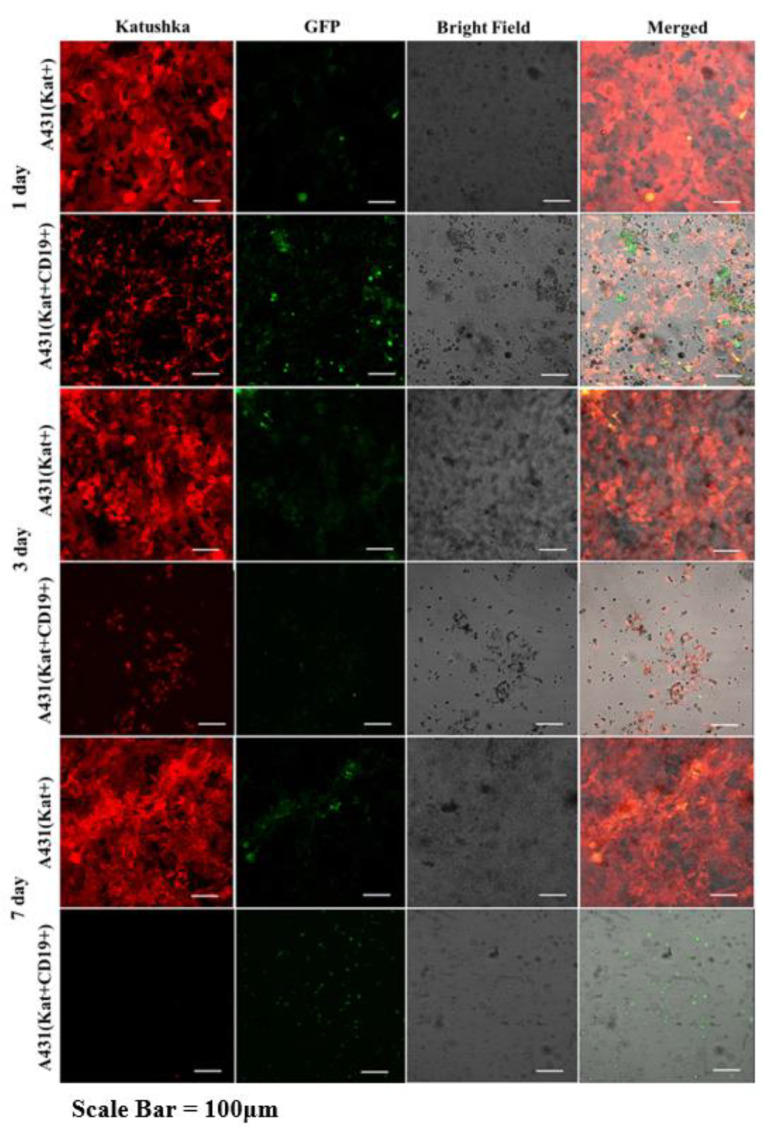
Micrographs of A431(Kat+) and A431(Kat+CD19+) tumor cell monolayers treated with CAR-T cells for a period of 7 days. Light microscopy, fluorescence microscopy, and representative micrographs (*n* = 6) are presented, scale bar is equal to 100 µm. CAR-T cells—green fluorescence; A431(Kat+) and A431(Kat+CD19+) cells—red fluorescence.

**Figure 14:**
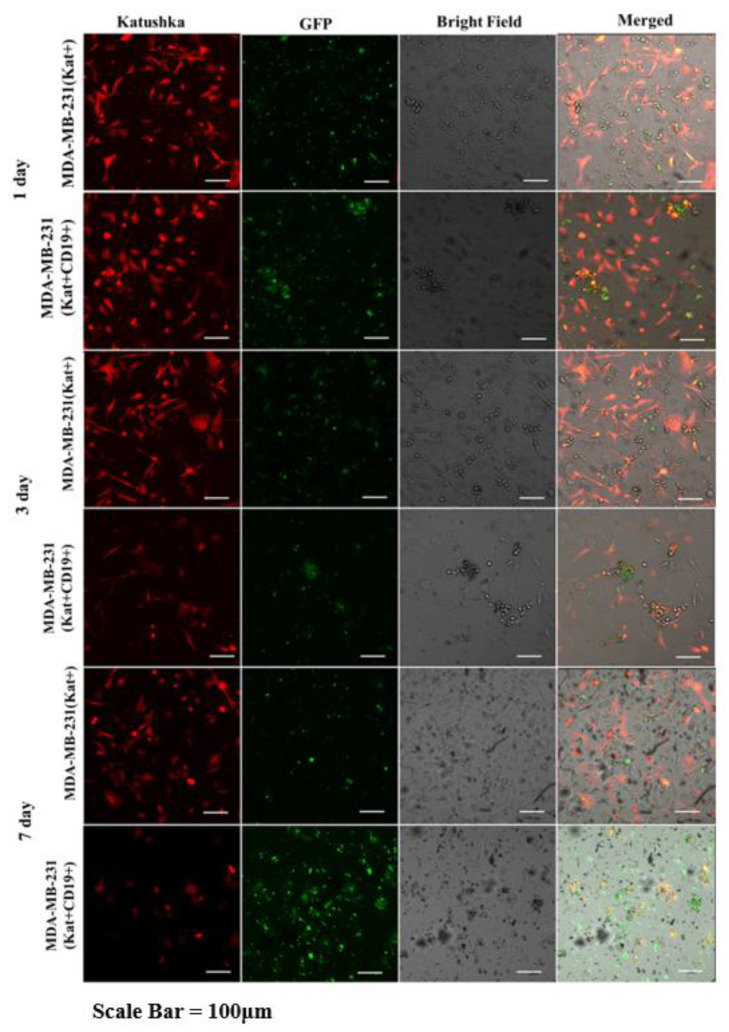
Micrographs of MDA-MB-231(Kat+) and MDA-MB231(Kat+CD19+) tumor cell line monolayers treated with CAR-T cells for a period of 7 days. Light microscopy, fluorescence microscopy, and representative micrographs (*n* = 6) are presented, scale bar is equal to 100 µm. CAR-T cells—green fluorescence; MDA-MB-231(Kat+) and MDA-MB-231(Kat+CD19+) cells—red fluorescence.

Fluorescence microscopy data (Figure 11, Figure 12, Figure 13 and Figure 14) are consistent with the expected CAR-T mediated granzyme/perforin cytolysis of the tumor cells. Normally CAR-T cells release granzymes and perforins upon recognition of target tumor cells, which further leads to a perforin-mediated formation of pores on the target cell membrane, thereby facilitating the passive diffusion of pro-apoptotic proteases, such as granzymes. Granzyme B is a serine protease that cleaves the pro-apoptotic molecule BID (BH3-interacting domain of the death agonist) resulting in the truncated form of tBID that induces apoptosis through the mitochondrial (internal) pathway or by direct activation of caspases 3 and 7 [9].

### 3.5. Evaluation of the Efficacy of CAR-T Cells against 3D Tumor Cell Cultures

**Figure 15:**
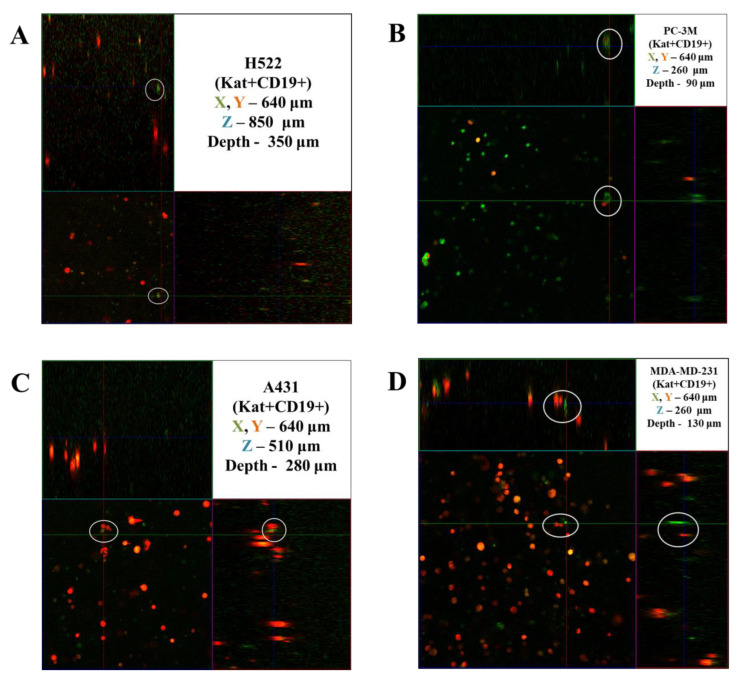
Orthotopic micrograph of 3D structures formed by tumor cells. (**A**)—H522(Kat+CD19+), (**B**)—PC-3M(Kat+CD19+), (**C**)—A431(Kat+CD19+), and (**D**)—MDA-MB-231(Kat+CD19+) tumor cells. Confocal fluorescence microscopy and representative micrographs are presented (*n* = 6). Circles indicate examples of single CAR-T cells (green fluorescence) and areas of their direct contact with tumor cells (red fluorescence).

The activity of CAR-T cells against 3D cultures of tumor cells was assessed by dynamic observation of tumor-like structures using a confocal scanning microscope LSM 700 (Carl Zeiss, Oberkochen, Germany) in 3D mode. Figure 15 shows the results of confocal microscopy analysis, representatively showing the penetration of CAR-T cells deep into three-dimensional tumor-like structures on the 4th day of co-culturing. The circles indicate areas of direct contact between CAR-T and tumor cells. CAR-T cells were able to penetrate three-dimensional tumor-like structures formed by H522(Kat+) and H522(Kat+CD19+), PC-3M(Kat+) and PC-3M(Kat+CD19+), A431(Kat+) and A431(Kat+CD19+), and MDA-MB-231(Kat+) and MDA-MB-231(Kat+CD19+). At the same time, CAR-T cells were located at different levels of the matrix depth, including in the immediate vicinity of tumor cells.

### 3.6. Evaluation of Cytokines and Chemokines in Supernatants via Multiplex Analysis

**Figure 16:**
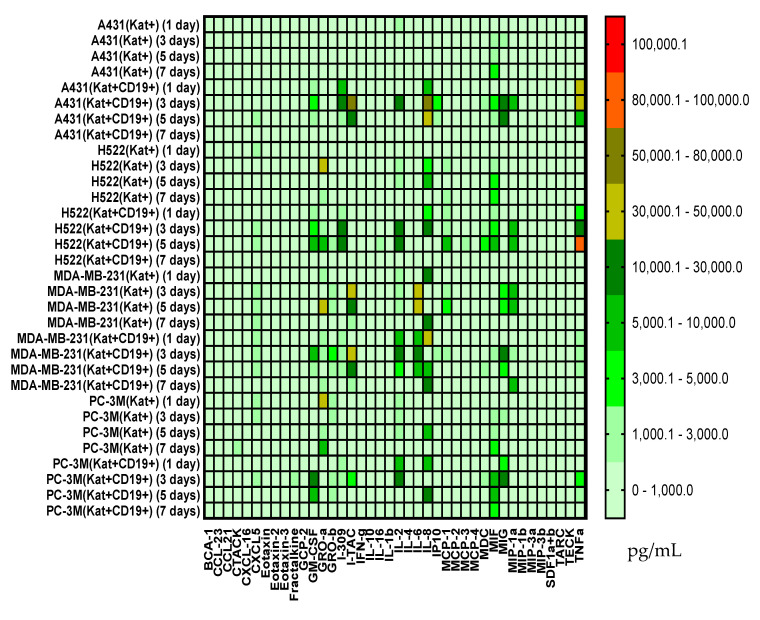
Heat map of cytokines and chemokines multiplex arrays in the culture medium after co-cultivation of CAR-T cells with H522(Kat+), H522(Kat+CD19+), PC-3M(Kat+), PC-3M(Kat+CD19+), A431(Kat+), A431(Kat+CD19+), MDA-MB-231(Kat+), and MDA-MB-231(Kat+C19+) tumor cells at different time points (days 1, 3, 5, and 7).

The co-incubation of CAR-T cells with H522(Kat+CD19+) cells led to increased secretion of GM-CSF on days 3 to 5 with a decrease on day 7; increased I-309 on days 3 to 5; increased MIF on days 3 to 5; increased TNFα on days 3 to 5 with a decrease on day 7 (Figure 16). Similarly, the co-incubation of CAR-T cells with PC-3M(Kat+CD19+) cells resulted in an increased secretion of IL-4 on days 1 to 3 after the addition of CAR-T cells with a subsequent decrease on days 5 to 7; increased MIF on days 3 to 5 with a decrease on day 7. The co-cultivation of CAR-T cells with A431(Kat+CD19+) resulted in increased levels of I-309 on days 1 to 3 after the addition of CAR-T cells; increased I-TAC on day 3; increased IL-8 on days 1 to 3 with a decrease on day 7; increased MIG on days 3 to 5; increased TNFα on days 1 to 3 with a gradual decrease on days 5 to 7. The co-cultivation of CAR-T cells with MDA-MB-231(Kat+CD19+) resulted in increased levels of TNFα on days 1 to 5; IL-2, IL-6, MIG, GM-CSF on days 3 to 5; IP10, MIF on day 3 (Figure 16). At the same time, for all samples, a decreased level of these cytokines was observed on day 7.

## 4. Discussion

Our experimental study demonstrated the cytotoxic effect of CAR-T cells against modified H522(Kat+CD19+), PC-3M(Kat+CD19+), A431(Kat+CD19+), and MDA-MB-231(Kat+C19+) cells and assessed the corresponding cytokine/chemokine profile of the intercellular interactions.

CAR-T cells were obtained with 86.98% of lentiviral transgene transduction efficiency. The results correlate well with those previously obtained by Pan et al., who also showed highly efficient transduction of T-lymphocytes [10]. This is in very good agreement with the previously reported data demonstrating that the percentage of CAR-positive cells within CAR-T cell products in clinical trials can vary from 20% to over 90% [11].

Real-time analysis of the proliferative potential of H522(Kat+CD19+), PC-3M(Kat+CD19+), A431(Kat+CD19+), and MDA-MB-231(Kat+C19+) cells using the xCELLigence biosensor system demonstrated a substantial decrease in the number of tumor cells after the addition of CAR-T cells, compared with the control non-modified H522 and PC-3M cells. Importantly, T cells did not have any significant impact on the progressive proliferation of H522(CD19+), PC-3M(CD19+), A431(Kat+CD19+), and MDA-MB-231(Kat+C19+) cells, suggesting no substantial off-target cytotoxicity. The obtained data indicate a partial cytolysis of CD19+ tumor cells and correlate with previously published results by Guedan et al., in which the effect of CAR-T cells on non–small cell lung carcinoma (L55) cells was assessed in real-time via the xCELLigence system [12]. Similar results were obtained by Yagyu et al. using the rhabdomyosarcoma Rh30 cell line that was co-cultivated with CAR-T and T cells [13]. Both CAR-T and T cells may be involved in two different pathways mediating tumor cell death: the perforin/granzyme pathway and the death receptor pathway. These signaling cascades share a common terminal effector pathway which leads to caspase-3 cleavage and finally triggers cellular apoptosis via DNA damage. Moreover, CAR-T and T cells may additionally secrete pro-inflammatory cytokines that act as effectors killing target tumor cells. The perforin/granzyme axis is considered to be the most important pathway for the cytotoxic functions of CD8+ and CD4+ T cells and, accordingly, for CAR-T cells [14]. However, recent evidence suggests that the death receptor axis and cytokine-mediated killing should not be overlooked when deciphering CAR-T cell-mediated tumor elimination [15].

We also performed a dynamic observation of a monolayer of H522(Kat+CD19+), PC-3M(Kat+CD19+), A431(Kat+CD19+), and MDA-MD-231(Kat+C19+) cells to assess the efficacy of CAR-T cells, and showed a significant progressive decrease in the confluence of CD19+ tumor cells. Petukhov et al. previously demonstrated that CAR-T cells dramatically reduce (down to 9.3%) the confluence of myelogenous leukemia (K562) cells transduced with the CD19 antigen [7]. At the same time, for control non-transduced tumor cells treated with CAR-T cells, the confluence remained practically unchanged at approx. 56.9.

Multiplex analysis showed an increase in the levels of some cytokines/chemokines upon incubation of CAR-T and tumor cells—GM-CSF, I-309, MIF, and TNFα for H522(Kat+CD19+) cells; IL-4 and MIF for PC-3M(Kat+CD19+) cells; IFNγ, I309, IL-2, IL-8, MIG, MIP1α, and TNFα for A431(Kat+CD19+) cells; and TNFα, IL-2, IL-6, MIG, and GM-CSF for MDA-MB-231(Kat+CD19+) cells. These data correlate well with the previously published results by Zhang et al. who showed that anti-CD19 CAR-T cells secrete a wide range of cytokines (including IL-1β, IL-6, IL-8, and IL-10) when co-incubated with Raja CD19+ cells [16].

The cytotoxicity of CAR-T cells against tumor cells’ monolayer was, overall, well correlated with the cytokine/chemokine profile. The antitumor effect of CAR-T cells was starting to be observed after day 5 of co-incubation, according to confocal microscopy (a decrease in the confluence of the tumor cell monolayer, data not included) and multiplex analysis of cytokine/chemokine levels. Interesting to note that according to the previously reported clinical data, CRS also manifests itself during the first week after the infusion of CAR-T cells into the patient and can last for the next 1–2 weeks [17].

Thus, our model approach can potentially be applicable to assess the efficacy of prospective CAR-T cell therapeutics against solid tumors. A rational future development of this study would envisage consistent complication of the cell models to achieve different levels of tumor tissue organization by generating in vitro tumor-like three-dimensional structures and xenograft animal models.

## 5. Conclusions

Adoptive cell therapy using genetically modified T-lymphocytes, in particular CAR-T cells, is one of the most promising novel approaches to personalized anticancer therapy entering wider clinical practice. Despite the overall success of this therapy for the treatment of hematological neoplasms, unresolved problems do not allow for achieving stable positive results for other oncological diseases—particularly solid tumors.

In this work, we demonstrated the ability of CAR-T cells to perform antigen-specific lysis of solid tumor cell lines, which confirms their potential general use for the treatment of non-hematological malignant neoplasms. The use of multiplex cytokine/chemokine analysis to assess the antitumor performance of CAR-T cells has expanded the understanding of the intracellular mechanisms of activity and improved the methodology of in vitro modeling, which includes a consideration of the potential side effects of CAR-T therapy, such as cytokine release syndrome. The results add further evidence suggesting that the overall low efficacy of CAR-T therapy against solid tumors might be conditioned by such tumor features as dense stroma or immunosuppressive microenvironment. Many current studies are aimed at understanding the mechanisms of the antitumor cellular response and preventing antigen escape, developing approaches to increase the survival and efficacy of CAR-T cells in the patient body. The results will expand the therapeutic value of CAR-T therapy and ultimately lead to the cure of many patients with previously incurable malignant neoplasms.

## Data Availability

Data sharing not applicable.
